# Impact of GSM-EMW exposure on the markers of oxidative stress in fetal rat liver

**DOI:** 10.1038/s41598-023-44814-z

**Published:** 2023-10-18

**Authors:** Mariam Salameh, Sukaina Zeitoun-Ghandour, Lina Sabra, Ahmad Daher, Mahmoud Khalil, Wissam H. Joumaa

**Affiliations:** 1 Rammal Hassan Rammal Research Laboratory, PhyToxE Research Group, Faculty of Sciences Section V, Lebanese University, Nabih Berri Street, Nabatieh, Lebanon; 2 Department of Biological Sciences, Faculty of Science, Beirut Arab University, Beirut, Lebanon; 3 Rammal Hassan Rammal Research Laboratory, ATAC Research Group, Faculty of Sciences (I), Lebanese University, Hadat, Lebanon; 4 Molecular Biology Unit, Department of Zoology, Faculty of Science, Alexandria University, Alexandria, Egypt

**Keywords:** Biochemistry, Molecular biology, Environmental sciences, Diseases, Health care

## Abstract

The current study investigated the effects of 24 h/day prenatal exposure to global system for mobile communication electromagnetic fields (GSM-EMFs), 900 MHZ-induced electromagnetic radiation (EMR), on oxidative stress (OS) status, apoptotic, and inflammatory changes in liver of rats during their fetal development period. Fifty-two *Sprague–Dawley* pregnant rats were equally divided into control and exposed groups. Whole embryos were removed at 7.5 dpc (days post coitus), while liver tissues were extracted from embryos at 11.5, 15.5, and 19.5 dpc. For exposed animals, results showed an increased OS reflected by high levels of malondialdehyde (MDA), a decrease in cytosolic superoxide dismutase (cytoSOD) activity, in mitochondrial superoxide dismutase (mitoSOD) levels and catalase (CAT) mRNA expression but also in hepatic nuclear factor erythroïd 2-related Factor 2 (Nrf-2), protein kinase B (Akt1), and intercellular adhesion molecule-1 (ICAM-1) mRNA expression at 15.5 dpc. Moreover, GSM-EMR exposure was shown to significantly decrease mitoSOD and CAT activities at almost all studied ages. Thus, rat embryos may be protected by their mothers from OS, apoptotic, and pro-inflammatory responses till a sensitive developmental stage, during a continuous prenatal EMR exposure. This protection could be then created from the embryos themselves.

## Introduction

The main function of the liver, the largest internal organ in the human body, is detoxification by eliminating all toxins^1^. The harmful effects of free radicals produced by hepatic metabolism could be mainly attenuated by the function of many antioxidant enzymes in addition to other non-enzymatic mechanisms^2,3^. However, a decrease in the antioxidant system response accompanied by excessive production of reactive oxygen species may increase the hepatic oxidative stress, and result in liver injury. Many autoimmune diseases, unhealthy lifestyles, and exposure to environmental pollutants such as electromagnetic radiation, could lead to oxidative stress^4^.

The sources of electromagnetic radiation (EMR) could be numerous including machines such as mobiles, and radars, in addition to phones’ base stations, electrical devices, and other technological apparatuses^5^. These radiations mainly affect the liver, kidneys, and brain because of their proximity to mobile phones during our daily use. Generally, the effects of these radiations can be determined according to the distance of their source or the duration of exposure at low and high frequencies^6^, in addition to, the widespread of mobile stations, radars, and basic daily dependence on mobile phones in all fields^7^. Moreover, exposure to these radiations leads to oxidative stress (OS) in body tissues. This phenomenon is a result of the imbalance between the levels of reactive oxygen species (ROS) and the ability of the antioxidant system to counter ROS effects^3^. Oxidative stress could also increase during various stages of development ^8^.

On the other side, EMR exposure during pregnancy and neonatal development of rats increased the susceptibility of the brain, more than the liver, to oxidative injury after exposure to 2.45 GHz Wi-Fi-induced electromagnetic radiations for 60 min/day for 5 days/week during the gestation period till three weeks of age^9^. The brain oxidative stress was reduced by the liver antioxidant capacity after exposure to mobile phone-induced electromagnetic radiation (900 and 1800 MHz) for 1 h/day from the pregnancy period till six weeks of age^10^. Unlike these previous reports that studied the effects of discontinuous intermittent electromagnetic fields (EMFs) exposure from diverse sources and frequencies at limited ages and organs, and as a complement to our recent study that have shown that a continuous (24 h/day) prenatal and postnatal 900 MHz GSM-EMR exposure may induce an increased oxidative stress status in liver of neonates and young female rats from their postnatal day 9 (PND9)^11^, we focused on the effects of continuous exposure to radiofrequency electromagnetic radiation (RF-EMR) of 900 megahertz (MHz) produced by mobile phone base stations antenna on whole embryos and hepatic tissues of rat embryos and fetuses aged 7.5, 11.5, 15.5, and 19.5 of their embryonic days or days post coitus (dpc), via exposing the mothers to these radiations during all gestation period. Therefore, this study investigated the effects of these electromagnetic radiations on oxidative stress, inflammatory, and apoptotic parameters in the liver of rat embryos and fetuses such as the malondialdehyde (MDA) level, the amount, relative activity, and gene expression of the most important anti-oxidation enzymes (SOD, GPx, and CAT), in addition to the alteration in the expression levels of Nrf-2, Akt1 and ICAM-1.

## Materials and methods

### Animals

Authors complied with the ARRIVE guidelines, all animal operations were carried out through National Institutes of Health (NIH) guidelines, and the animal preparation protocol was approved by the Institutional Review Board (IRB) committee at Beirut Arab University, Beirut, Lebanon. In each time, four *Sprague–Dawley* female rats and one male were housed together in a plexiglass cage for mating. Vaginal plugs were examined every morning between 8:30 and 10:30 am as a sign of mating^12^. The size of the litter was adjusted to obtain litters of the equivalent size and only pregnant female rats were used in this study. All pregnant rats were at room temperature (22–23 °C), on a 12:12 light–dark cycle in a controlled chamber.

### Radiation exposure

**Figure 1:**
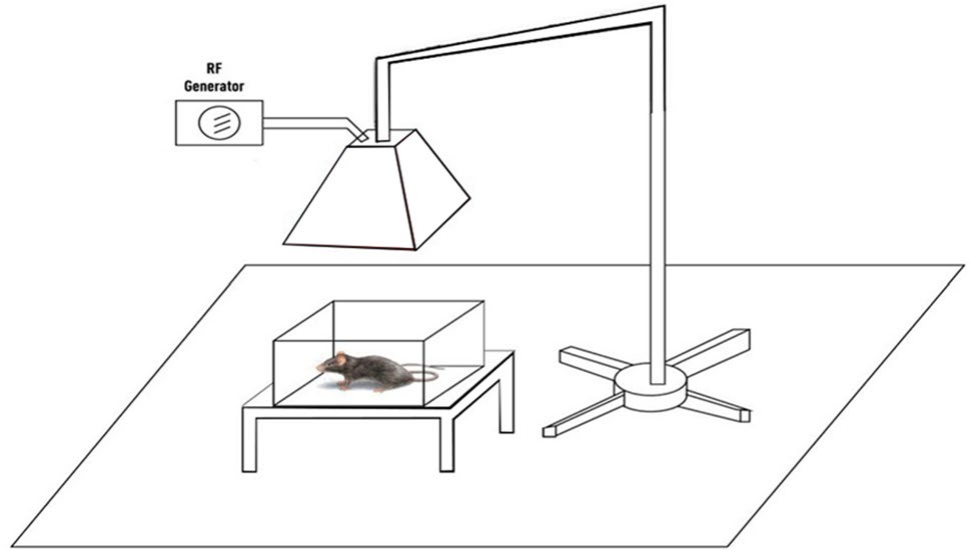
Global system for mobile communication radiofrequency electromagnetic radiation GSM-(RF-EMR) exposure system.

Pregnant female rats were exposed for 24 h per day to RF-EMR of type GSM (900 megahertz of frequency, Eeff equal to 25 ± 0.4 V/m) from the first gestational day till the day of sacrifice. Rats received this radiation across their whole body as shown by Salameh et al., and Ramadan et al.^11,13^ using an apparatus similar to the mobile phone base stations antenna. This device generally consists of a radiofrequency signal (RF) generator (model RFS 900–64, RFPA, Artigues-près-Bordeaux, France) accompanied by RF-EMF antenna which is supported by a stand, and placed above the cages containing rats (Fig. 1). These cages were of 55 cm long, 35 cm wide, and 15 cm high, and were distanced 100 cm from the RF antenna. To control the level of exposure to the radiofrequency electromagnetic radiations during the gestation period (gestational day 1, 7.5, 11.5, 15.5, and 19.5 dpc), a radiofrequency probe (PMM EP600, Narda Safety Test Solution, Hauppauge, NY, USA) monitored with computer software (Win EP 600, Narda Safety Test Solution) has been used. The specific absorption rate (SAR) in liver tissue (0.768 W/Kg) was estimated by using the following equation: SAR = σ*E^2^/ρ, where (σ) is the conductivity (1.34 S/m), (E) is the magnitude of the electric field (25 V/m), and (ρ) is the mass density of the tissue-equivalent media (1090 kg/m3)^14^.

### Animal groups

Pregnant rats were divided into two groups: control and exposed rats. Measurements were made on 26 control pregnant female rats in parallel with 26 exposed pregnant rats. Every two or three pregnant female rats were placed in a cage. The maternally- exposed and unexposed rat embryos were included in our study. Whole rat embryos were removed at 7.5 dpc (days post coitus), while their livers were extracted at 11.5, 15.5, and 19.5 dpc, from both the control and exposed animals. The embryonic day 7.5 represents nearly the end of the ‘Gastrula’ stages, 11.5 dpc represents the beginning of the ‘Embryo’ formation stage, 15.5 dpc is the end of the embryo metamorphosis period, and 19.5 dpc represents the second fetal stage. Mother rats were euthanized with an intraperitoneal sodium pentobarbital overdose (1 ml/kg; 200 mg/ml solution). Livers were flash-frozen in liquid nitrogen and then stored at − 80 °C for later experiments.

### Biochemical and molecular analysis

#### Determination of the lipid peroxidation product (MDA) levels

MDA level was detected in all rat embryos and rat fetuses’ livers at different stages (7.5 dpc, 11.5 dpc, 15.5 dpc, and 19.5 dpc) from both control and exposed pregnant mothers to EMR. This detection was made by using the lipid peroxidation (MDA) Colorimetric/Fluorometric Assay Kit from BioVision USA (100 assays, catalog # K739-100) according to the instructions of the manufacturer.

#### Measurement of superoxide dismutase (SOD) activity from mitochondria and cytosol fractions

To measure SOD activity from mitochondria and cytosol fractions separately, the Mitochondrial/Cytosol Fractionation Kit from BioVision (Catalog number: K256-25) was used to separate both hepatic fractions, and the Superoxide Dismutase (SOD) Activity Assay Kit from BioVision (100 reactions and a catalog number of K335-100) was then used to measure this enzyme activity (in U/mL) separately.

#### Measurement of glutathione peroxidase (GPx) activity

GPx activity was determined (in nmol/min/ml or mU/ml) in samples from control and exposed rats using the Glutathione Peroxidase Activity Colorimetric Assay Kit from BioVision USA (catalog # K762-100) according to the specified instructions. The detection sensitivity of GPx in samples is 0.5 mU/mL.

#### Measurement of catalase (CAT) activity

CAT activity (in mU/ml or nmol/min/ml) was measured in samples from control and exposed rats using the Catalase Activity Colorimetric/Fluorometric Assay Kit from BioVision USA (catalog # K773-100) according to the specified instructions.

#### Estimation of the relative enzyme activities

The quantification of the enzymes was done by Bradford reagent (Sigma ALDRICH, B6916), where it’s possible to quickly estimate the protein concentration in a small volume (5 µl) of studied samples. The measurement of absorbance was made at a wavelength equal to 595 nm, and the protein dosage is determined in mg/ml of the original solution and then in mg/g of liver mass. Relative activity was calculated from the absolute enzymatic activity and the protein dosage of the enzymes for the antioxidant work in each of the measured samples. The activity of each enzyme (GPx, CAT, and SOD) measured in mU/ml, was divided by the duration of the experimental reaction (in min), and then divided by the protein concentration measured by the Bradford reagent (in mg/ml). The relative activity in mU/min/mg was converted to another common unit which is μmol/min/mg of protein, divided by a factor of 1000.

#### Determination of SOD1, GPx1, CAT, Nrf-2, ICAM-1 and Akt1 mRNA levels using quantitative Real-Time PCR (q-RT-PCR) technique

Total RNA was extracted from rat embryos and hepatic tissues of rat fetuses by using the Quick-RNA TM MiniPrep Plus Kit (ZYMO RESEARCH, catalog nos. R1057 and R1058). RNA samples were transcribed into cDNA using the iScript™ cDNA Synthesis Kit (Bio-RAD, USA, catalog number: 1708891). Specific primers purchased from Macrogen, Korea (shown in Table 1) were used for RT-PCR to determine the levels of the steady-state mRNA of desired proteins (SOD1, GPx1, CAT, Nrf-2, ICAM-1, and Akt1). The iTaq™ Universal SYBR ® Green Supermix (Bio-Rad, USA, catalog number: MLL4801) was used in this quantitative detection. Real-time PCR reactions were performed in triplicates by using a thermal cycler with a CFX Connect Real-Time PCR Detection System (BIO-RAD, USA, catalog number: 1855200). The comparative CT method which depends on the value of the studied genes to the reference gene was used to calculate the fold difference in gene expression (2^−ΔΔCt^ ) with beta-actin (β-Actin) and TATA box binding protein (TBP) as reference genes and control untreated samples as calibrator (2^−ΔΔCt^ = 1).

**Table 1:** Specific forward and reverse primers (From macrogen) designed for oxidative stress, inflammation and apoptosis-related genes (SOD1, GPx1, CAT, Nrf-2, ICAM-1 and Akt1), and for reference endogenous genes (ß- Actin and TBP) in rat liver.

Symbols	Genes	Primer Sequences
	*Rattus norvegicus*	(Forward and Reverse): 5′ → 3’
SOD1	Superoxide dismutase 1	F: CCACTGCAGGACCTCATTTT
		R: CACCTTTGCCCAAGTCATCT
GPx1	Glutathione peroxidase 1	F: ATAGAAGCCCTGCTGTCCAA
		R: GAAACCGCCTTTCTTTAGGC
CAT	Catalase	F: ACATGGTCTGGGACTTCTGG
		R: CAAGTTTTTGATGCCCTGGT
Nrf-2	Nuclear factor-erythroid derived 2-like 2	F: CCTAAAGCACAGCCAACACA
		R: GCCTCTAATCGGCTTGAATG
ICAM-1	Intercellular adhesion molecule 1	F: AGGTATCCATCCATCCCACA
		R: GCCACAGTTCTCAAAGCACA
Akt1	Protein kinase B	F: CCTCAAGAATGATGGCACCT
		R: TTTGAGTCCATCAGCCACAG
ß-Actin	ß-Actin	F: GGGTATGGAATCCTGTGGCATCC
		R: GCTCAGGAGGAGCAATGATCTTGA
TBP	TATA box binding protein	F: GACTCCTGTCTCCCCTACCC
		R: CTCAGTGCAGAGGAGGGAAC

### Western blotting technique

Liver tissue was collected from rat fetuses at the stage of 19.5 dpc of the prenatal embryonic period and then homogenized in a lysis extraction buffer. Extracted proteins were quantified with Lowry protein essay at 750 nm wavelength. Western blot technique was performed as described previously^15^. In brief, samples were loaded and total proteins were separated on sodium dodecyl sulfate (SDS) acrylamide gels before being electro-transferred to nitrocellulose membranes. Membranes were then washed several times with 1 × DPBS (SIGMA ALDRICH) before being incubated overnight at 4 °C with gentle agitation in specific primary antibodies: anti- Nrf-2 (ab31163, Abcam, USA), and anti- GAPDH as a loading control. Protein bands were detected using the Clarity™ Western ECL Substrate Kit (Bio-rad, USA, Cat #: 170-5060), standardized to GAPDH levels, and quantified by the Image-J. analysis software.

### Statistical analysis

Values were presented as means ± SEM for n observations. Mann–Whitney U test was applied to compare the changes in parameters between the control and exposed groups at the different ages of testing. Normality measure was performed by using the Kolmogorov–Smirnov test. Statistical studies were performed in SPSS software (version 20, SPSS Inc., Chicago, U.S.A.). *P*-values < 0.05 represented a significant difference.

### Ethics approval

All animal operations in the study were performed through National Institutes of Health (NIH) guidelines. Animal preparation protocol was approved by the Institutional Review Board (IRB) committee at Beirut Arab University, Beirut, Lebanon.

## Results

### Effect of GSM-EMW prenatal exposure on hepatic MDA level

**Figure 2:**
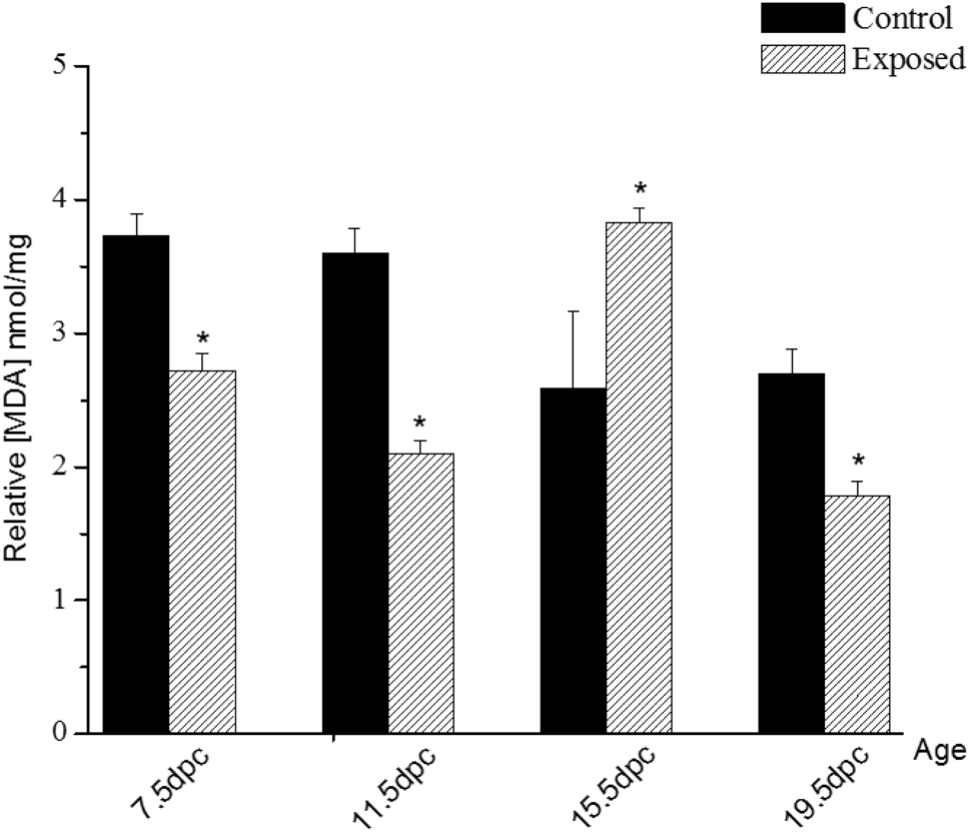
Effects of EMR exposure on the level of malondialdehyde (MDA) product (as mean ± SEM) in nmol/mg of the control and exposed whole embryos aged of 7.5 dpc, and liver tissue of the control and exposed groups of rat embryos and fetuses aged of 11.5, 15.5, and 19.5 of their embryonic days or days post coitus (dpc) (n = 12), (P-value < 0.05 (*) represents a significant change in results for exp. vs. ctrl. group).

To assess oxidative stress in certain pathophysiological processes, it is primary to quantify the level of lipid peroxidation products such as MDA. Compared to the control group, the exposure to EMR showed a significant reduction (*P* < 0.001; n = 12) of nearly 27%, 42%, and 34% in mean MDA level in the exposed group at the embryonic ages 7.5 dpc, 11.5 dpc, 19.5 dpc, respectively, but a significant elevation (*P* = 0.039; n = 12) of nearly 49% at 15.5 dpc (Fig. 2).

### Effect of GSM-EMW prenatal exposure on hepatic mitochondrial and cytosolic SOD

SOD is one of the most important antioxidant enzymes that may be studied. It catalyzes the process of superoxide anions dismutation into hydrogen peroxide (H_2_O_2_) and molecular water.

By comparing repeated measures of SOD mitochondrial fraction, it was shown that in comparison with the control group, no significant change (*P* = 0.05281) of this fraction was observed in whole exposed rat embryos at 7.5 dpc. Then, a significant increase of hepatic SOD mitochondrial fraction (*P* < 0.05; *P* < 0.00001) by approximately 62% and 122% was detected in exposed rat embryos and fetuses at 11.5 dpc and 19.5 dpc, respectively. But a significant reduction (*P* = 0.000175) by nearly 69% was observed in those aged 15.5 dpc (Table 2).

**Table 2:** Amounts of mitochondrial superoxide dismutase (mitoSOD) protein fraction as mean ± SEM in mg/g of control and exposed whole embryos aged of 7.5 dpc, and liver tissues of control and exposed groups of rat embryos and fetuses aged of 11.5, 15.5, and 19.5 of their embryonic days or days post coitus (dpc) (Ctrl: control group; SAR: specific absorption rate). (P-value < 0.05 (*) represents a significant difference in results for exposed versus control group).

Group	Age (dpc)	N	SOD mitochondrial protein fraction (mg/g of liver mass)
Ctrl	7.5	18	0.768 ± 0.09
SAR (0.768 W/Kg)	7.5	12	1.01 ± 0.05
Ctrl	11.5	18	3.117 ± 0.617
SAR (0.768 W/Kg)	11.5	12	5.039 ± 0.317*
Ctrl	15.5	24	1.619 ± 0.267
SAR (0.768 W/Kg)	15.5	24	0.505 ± 0.056*
Ctrl	19.5	24	0.57 ± 0.086
SAR (0.768 W/Kg)	19.5	24	1.263 ± 0.1*

Compared to unexposed groups, a non-significant change (*P* > 0.05) was detected in the mean SOD cytosolic fraction in whole exposed rat embryos at 7.5 dpc, and of the hepatic SOD cytosolic fraction in exposed rat embryos aged of 11.5 dpc, respectively. However, a significant increase (*P* < 0.00001) by ~ 761% and a significant reduction (*P* < 0.00001) of ~ 58% in mean hepatic cytoSOD were then detected in exposed embryos and fetuses aged 15.5 dpc and 19.5 dpc, respectively (Table 3).

**Table 3:** Amounts of cytosolic superoxide dismutase (cytoSOD) protein fraction as mean ± SEM in mg/g of control and exposed whole embryos aged of 7.5 dpc, and liver tissues of control and exposed groups of rat embryos and fetuses aged of 11.5, 15.5, and 19.5 of their embryonic days or days post coitus (dpc) (Ctrl: control group; SAR: specific absorption rate). (P-value < 0.05 (*) represents a significant difference in results for exposed versus control group).

Group	Age (dpc)	N	SOD cytosolic protein fraction (mg/g of liver mass)
Ctrl	7.5	18	0.268 ± 0.0113
SAR (0.768 W/Kg)	7.5	18	0.256 ± 0.010
Ctrl	11.5	36	0.149 ± 0.007
SAR (0.768 W/Kg)	11.5	24	0.124 ± 0.0157
Ctrl	15.5	24	0.018 ± 0.003
SAR (0.768 W/Kg)	15.5	24	0.155 ± 0.022*
Ctrl	19.5	36	0.112 ± 0.008
SAR (0.768 W/Kg)	19.5	24	0.047 ± 0.007*

### Effect of GSM-EMW prenatal exposure on hepatic GPx and catalase protein levels

Glutathione peroxidase (GPx) is a family of antioxidant enzymes where various isozymes were founded in cells with different substrate specificity. Glutathione peroxidase reduces free hydrogen peroxide to water or lipid hydroperoxide to alcohols while oxidizing reduced glutathione (GSH) to oxidized glutathione (GSSG). The generated GSSG is converted to GSH by glutathione reductase with the consumption of NADPH. Compared to control ones of the same age, EMR exposure was shown to significantly decrease (*P* < 0.0001) the mean hepatic GPx protein level in exposed rat embryos by nearly 45% at 11.5 dpc. However, in comparison to the control group, no significant change (*P* > 0.05) in mean GPx protein level was observed in whole exposed rat embryos at 7.5 dpc and in the liver tissue of exposed rat embryos and fetuses aged of 15.5 dpc and 19.5 dpc (Table 4).

**Table 4:** Glutathione peroxidase (GPx) protein levels as mean ± SEM in mg/g of control and exposed whole embryos aged of 7.5 dpc, and liver tissues of control and exposed groups of rat embryos and fetuses aged of 11.5, 15.5, and 19.5 of their embryonic days or days post coitus (dpc) (Ctrl: control group; SAR: specific absorption rate). (*P*-value < 0.05 (*) represents a significant difference in results for exposed versus control group).

Group	Age (dpc)	N	GPx protein level (mg/g of liver mass)
Ctrl	7.5	22	8.134 ± 0.860
SAR (0.768 W/Kg)	7.5	22	8.98 ± 1.05
Ctrl	11.5	22	15.852 ± 0.581
SAR (0.768 W/Kg)	11.5	24	8.679 ± 0.486*
Ctrl	15.5	20	10.676 ± 0.691
SAR (0.768 W/Kg)	15.5	18	13.34 ± 1.738
Ctrl	19.5	20	13.722 ± 1.589
SAR (0.768 W/Kg)	19.5	22	15.91 ± 2.09

Catalase (CAT) is an antioxidant enzyme that is nearly present in all living organisms. It helps in the reaction of decomposition of hydrogen peroxide into oxygen and water. After exposure to electromagnetic radiation, and in comparison with the control group, statistical tests showed a significant increase (*P* < 0.00001; *P* < 0.00001; *P* = 0.01107) in the mean CAT protein level of ~ 62%, 62%, 67% in whole exposed rat embryos aged 7.5 dpc, in hepatic tissue of exposed embryos aged 11.5 dpc and 15.5 dpc respectively, but a significant reduction (*P* = 2.64E-6) of ~ 43% in hepatic tissue of exposed fetuses aged of 19.5 dpc (Table 5).

**Table 5:** Catalase (CAT) protein levels (as mean ± SEM) in mg/g of control and exposed whole embryos aged of 7.5 dpc, and liver tissue of control and exposed groups of rat embryos and fetuses aged of 11.5, 15.5, and 19.5 of their embryonic days or days post coitus (dpc) (Ctrl: control group; SAR: specific absorption rate). (P-value < 0.05 (*) represents a significant change in results for exposed vs. control group).

Group	Age (dpc)	N	CAT protein level (mg/g of liver mass)
Ctrl	7.5	22	7.578 ± 0.401
SAR (0.768 W/Kg)	7.5	24	12.273 ± 0.537*
Ctrl	11.5	18	10.87 ± 1.11
SAR (0.768 W/Kg)	11.5	24	17.617 ± 1.07*
Ctrl	15.5	22	11.55 ± 0.897
SAR (0.768 W/Kg)	15.5	19	19.327 ± 1.445*
Ctrl	19.5	22	14.950 ± 1.144
SAR (0.768 W/Kg)	19.5	24	8.582 ± 0.559*

### Evaluation of the relative enzyme activity of studied antioxidant enzymes

#### Relative superoxide dismutase (SOD) activity

##### SOD mitochondrial fraction

**Figure 3:**
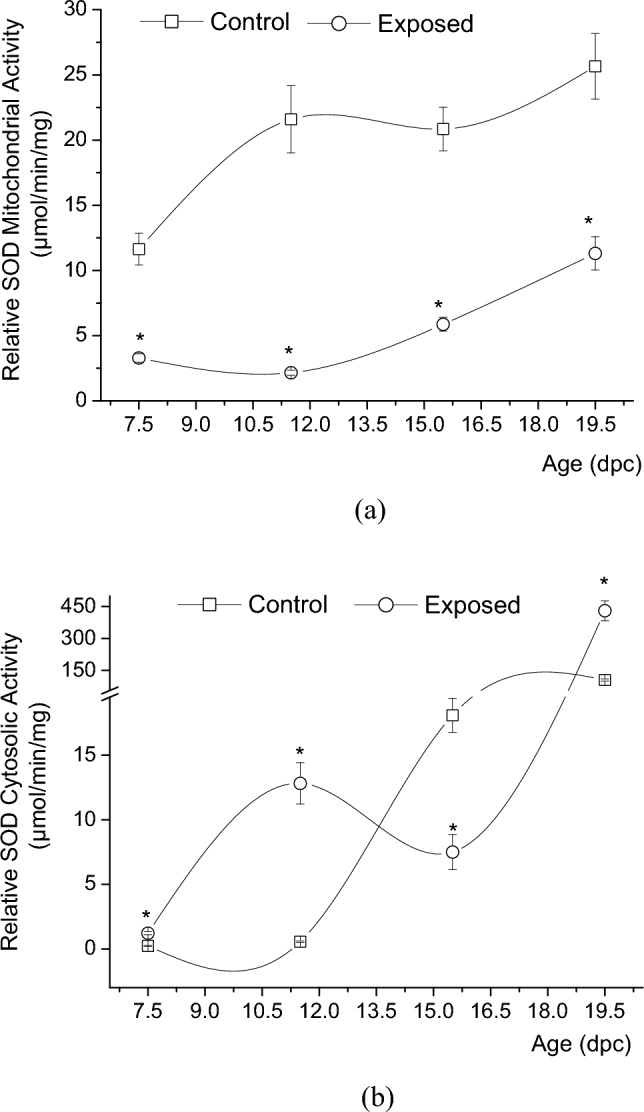
Measurements of the (**a**) relative superoxide dismutase (SOD) mitochondrial activity, and the (**b**) relative superoxide dismutase (SOD) cytosolic activity, presented as mean ± SEM (in µmol/min/mg of protein) in whole embryos and hepatic tissues, in both the control and exposed groups of rat embryos and fetuses aged of 7.5 (n = 36, n = 32; n = 18, n = 18), 11.5 (n = 36, n = 32; n = 36, n = 24), 15.5 (n = 48, n = 48; n = 5, n = 24), and 19.5 (n = 48, n = 48; n = 63, n = 48) of their embryonic days or days post coitus (dpc), (*P*-value < 0.05 (*) represents a significant change in results for exp. vs. ctrl. group).

GSM-EMW exposure was shown to significantly reduce by approximately 72%, 90%, 72%, and 56%, the mean of mitochondrial SOD relative activity in exposed rat embryos and fetuses in comparison with the control ones, at the four embryonic studied ages 7.5 dpc (n = 36; n = 32; *P* < 0.001), 11.5 dpc (n = 36; n = 32; *P* = 0.003), 15.5 dpc (n = 48; n = 48; *P* < 0.001), and 19.5 dpc (n = 48; n = 48; *P* < 0.001), respectively (Fig. 3a).

#### SOD cytosolic fraction

The exposure to EMR was shown to significantly increase the mean of cytosolic SOD relative activity by approximately 432%, 2193%, and 313% at the embryonic ages 7.5 dpc (n = 18; n = 18; *P* < 0.0001), 11.5 dpc (n = 36; n = 24; *P* = 0.00167), and 19.5 dpc (n = 63; n = 48; *P* < 0.0001), respectively, compared with the control ones. However, this relative activity was shown to be significantly reduced by nearly 59% in exposed embryos aged 15.5 dpc (n = 5; n = 24; *P* = 0.00188), when compared to those of the control group at the same age (Fig. 3b).

#### Relative glutathione peroxidase (GPx) and catalase (CAT) activities

**Figure 4:**
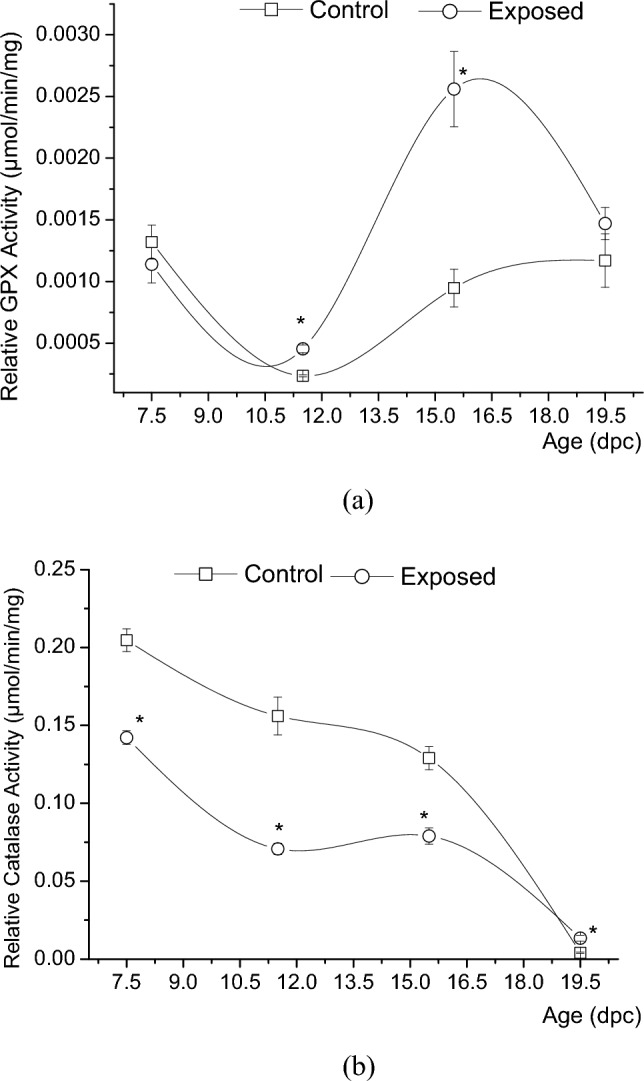
Measurements of the (**a**) relative glutathione peroxidase (GPx) activity, and the (**b**) relative catalase (CAT) activity, presented as mean ± SEM (in µmol/min/mg of protein) in whole embryos and liver tissues, in both the control and exposed groups of rat embryos and fetuses aged of 7.5 (n = 48, n = 48; n = 48, n = 48), 11.5 (n = 48, n = 48; n = 48, n = 48), 15.5 (n = 48, n = 48; n = 48, n = 48), and 19.5 (n = 48, n = 48; n = 39, n = 48) of their embryonic days or days post coitus (dpc), (*P*-value < 0.05 (*) represents a significant change in results for exp. vs. ctrl. group).

EMR exposure was shown to induce a significant increase (n = 48; n = 48; *P* < 0.001) of GPx relative activity, in rat embryos and livers, by nearly 93%, 170% at the embryonic age 11.5 dpc and 15.5 dpc, respectively. However, no significant change (n = 48; n = 48; *P* > 0.05) in mean GPx relative activity was shown in the exposed group at the baseline age of 7.5 dpc and 19.5 dpc as shown in Fig. 4a. Repeated measurements for relative catalase activity in rat embryos and livers showed a significant reduction (n = 48; n = 48; *P* < 0.001) of nearly 31%, 54%, and 39%, but a significant increase (n = 39; n = 48; *P* < 0.001) by nearly 225% at the four embryonic tested ages 7.5 dpc, 11.5 dpc, 15.5 dpc, 19.5 dpc, respectively (Fig. 4b).

#### Effect of GSM-EMW prenatal exposure on SOD1, GPx1, Catalase, and Nrf-2 mRNA expression

With comparison to the control group, it was noticed that SOD1 mRNA expression significantly decreased in the exposed samples to about 0.7% (0.00737 ± 1.539E-5), 5% (0.04917 ± 0.00433), and 49% (0.49318 ± 0.08126) at the embryonic ages 7.5 dpc, 11.5 dpc, and 19.5 dpc, respectively. While it significantly increased approximately 17 times (17.07544 ± 1.14134) at 15.5 dpc (*P* < 0.05). For GPx1, the EMR exposure was shown to significantly decrease its mRNA expression to nearly 85% (0.84787 ± 0.0938) at the first embryonic studied age of 7.5 dpc and significantly increase this expression to approximately double (2.27203 ± 0.29887), and the triple (2.65773 ± 0.24295) at 11.5 dpc and 15.5 dpc, respectively, with no significant changes at 19.5 dpc (1.12 ± 0.19169). Moreover, in comparison to the control rats, CAT mRNA expression showed a fluctuation among ages. A significant decrease to about half (0.53 ± 0.09), a significant increase to approximately 9 times (9.19 ± 1.87), a significant re-decrease to nearly 1% (0.0105 ± 0.003), followed by another re-increase of about 2.5 times (2.47665 ± 0.48) was detected at the four studied prenatal ages respectively: 7.5 dpc, 11.5 dpc, 15.5 dpc, and 19.5 dpc, in the exposed animals (*P* < 0.05). Regarding Nrf-2, an essential transcription factor leading to the up-regulation of many antioxidant genes expression and a sensor of oxidative stress status, its mRNA expression appeared persistently downregulated with 8% (0.08081 ± 0.00786), 44% (0.43973 ± 0.02711), 4% (0.04369 ± 0.0173), and 37% (0.37369 ± 0.05434) reduction in the different exposed studied age groups respectively (*P* < 0.05).

#### Effect of GSM-EMW prenatal exposure on Akt1 and ICAM-1 mRNA expression

Compared to the control group, the mRNA expression of Akt1 was significantly elevated in the exposed samples to approximately 1.4 times (1.38719 ± 0.17969), 8 times (8.12681 ± 1.34003), and 2.25 times (2.24946 ± 0.10331), at 7.5 dpc, 11.5 dpc, and 19.5 dpc, respectively. While it showed a significant decrease to approximately 68% (0.6824 ± 0.07798) at 15.5 dpc (P < 0.05).

However, the mRNA expression of ICAM-1 was shown to significantly being decreased in the exposed samples, to approximately 5.E-6% (5.1E-8 ± 5.3E-9), 15% (0.146 ± 0.014), and 7.E-2% (6.68E-4 ± 9.38E-5) at the first three studied ages (7.5 dpc, 11.5 dpc, and 15.5 dpc), but it significantly increased by about 5 times (4.881 ± 0.719) at the late embryonic age (19.5 dpc) (*P* < 0.05).

#### Effect of GSM-EMW prenatal exposure on Nrf-2 protein expression

**Figure 5:**
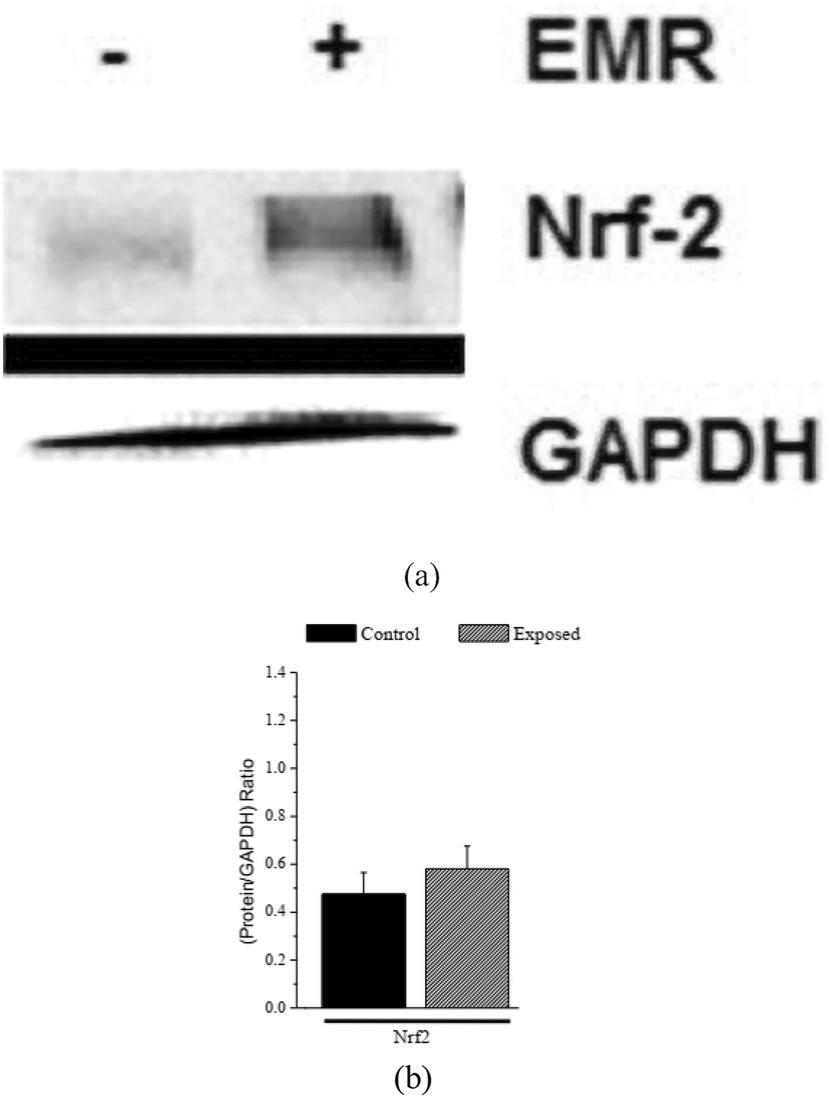
Effects of electromagnetic radiation (EMR) exposure on nuclear factor erythroid-2-related factor 2 (Nrf-2) protein expression. (**a**): western blotting results for Nrf-2, and Glyceraldehyde 3-phosphate dehydrogenase (GAPDH) standard protein in the liver of rat fetuses (n = 3) at 19.5 dpc in both the control (-) and the exposed group ( +). (**b**): A schematic presentation of the relative protein expression of Nrf-2 (ratio: protein/GAPDH). The presented values are the means ± SEM (*P*-value < 0.05 (*) represents a significant difference in the results for the exposed vs. the control group). (N.B: Original western blotting gels results for nuclear factor erythroid-2-related factor 2 (Nrf-2) and glyceraldehyde 3-phosphate dehydrogenase (GAPDH) were added as Fig. 5 (original) (a and b) in the Supplementary Information File).

The effects of exposure to EMW on Nrf-2 protein expression in rat fetuses’ livers were assessed by the western blot technique. The “19.5 dpc” late prenatal age represents the second fetal stage during development. The relative protein expression was quantified by using the Image J. analysis software and was standardized to GAPDH levels (ratio of protein/GAPDH). Results showed that GSM-EMW exposure has no significant effect on the Nrf-2 protein expression (0.58194 ± 0.18826; *P* > 0.05) in liver tissues of rat fetuses at 19.5 dpc when compared to the control animals at the same age (0.47538 ± 0.1807) (Fig. 5a,b) (Fig. 5(original) (a and b)).

## Discussion and conclusion

Based on the increasingly widespread use of mobile communication systems, a serious scientific discussion is being produced nowadays after following up on the effects of the exposure to radiation emitted from mobile phones and base stations on human health^16–18^. Cellular phones are major sources of electromagnetic radiation (EMR). The effects of these radiations were assessed in general depending on their intensity, time, distance of exposure^6^, and frequency^19^. EMR emitted from mobile phones may increase the production of reactive oxygen species, and therefore cause an oxidative stress status in different organs, especially the liver^19,20^. Electromagnetic waves may also exert an impact on human reproduction by affecting the development of embryos and fetuses^21^. The present study aimed to determine the effects of the permanent prenatal exposure of rat embryos and fetuses to 900 MHz GSM-EMFs radiation, emitted from mobile phones base stations antennas, on their hepatic oxidative stress, apoptotic and inflammatory responses, during the fetal development period. An increased oxidative stress level, defined as an imbalance status between the oxidative and anti-oxidative systems in cells and tissues, is generally caused by the overproduction of oxidative-free radicals and associated reactive oxygen species which could alter diverse proteins and lipids structures and functions leading to cellular dysfunctions^22^. Increased oxidative stress status may be caused by many physiopathological and physiological conditions^4,23^, nutritional causes^22^, and also from exposure to different external pollutants such as electromagnetic radiation^4^. MDA, a lipid peroxidation end product, is useful for the quantification of oxidative damage^24^ and is considered the main and most frequently measured biomarker of oxidative stress^25^. MDA level was shown to be higher in neonates of cesarean delivery^26^, in newborns with congenital anomaly^27^, in coronary artery disease^28^, hyperbilirubinemia^29^ cases, in high blood pressure^30^, after bisphenol A (BPA) exposure^31^, in embryos with hyperglycemia and hypoxia^32^, and in prenatal and postnatal exposed female rats to GSM-EMR from their PND 9^11^. In addition, MDA was measured in the brain, kidneys, and liver^2^. In offspring, MDA was shown to be at a higher level^33,34^ after a short^33^ or long exposure^35^ to an electromagnetic field. Our current results, compared to the control group, showed that the hepatic MDA level was higher in the exposed embryos aged 15.5 dpc during the fetal development period. However, reduced levels were detected in early development ages (7.5 dpc and 11.5 dpc), and late embryonic studied age (19.5 dpc) in the exposed ones. This result contradicts a previous study that showed a significant increase in MDA content in fetal rat brain on the 21^st^ day after exposure of mothers for a certain period during pregnancy to microwave radiation from cellular phones^36^. Here, the changes in the MDA level in the exposed group have been accompanied by significant changes in the activity of the antioxidant enzymes: SOD, catalase, and GPx. Thus, the decrease in MDA level in the exposed group, compared to the control one, at the first two embryonic ages (7.5 dpc and 11.5 dpc) may be more related to the increase in cytoSOD relative activity regardless of the significant decrease in mitoSOD and catalase relative activities. In addition, the significant increase in MDA level at 15.5 dpc could be explained by the high production of free radicals, and also related to the significant decrease in cytoSOD activity^37,38^ and other antioxidant enzymes except for GPx^38^. Moreover, despite the insignificant changes in GPx activity, and the permanent significant decrease in mitoSOD relative activity, the significant reduction in MDA level in the exposed group at the last studied age (19.5 dpc) could be possibly related not only to the increase of cytoSOD but also to catalase relative activity. Previous studies showed a negative relationship between gene expression and the production rate of proteins where a high production amount is shown to be a cause of a low level of gene expression^39^. Furthermore, an enzyme’s level seems to be proportionally related to its activity for a certain stage^40^. In the current study, these relationships were shown to be only applied for catalase antioxidant enzyme mRNA expression and its production rate at all studied embryonic ages except 11.5 dpc. A low catalase mRNA expression was detected with a high protein production rate at 7.5 dpc and 15.5 dpc, while a high mRNA expression level with a reduced protein production rate have been observed at 19.5 dpc. In addition, similar changes to the previous findings were shown in the directly proportional relationship between the enzyme level and its activity for GPx and mitochondrial SOD enzymes at the embryonic ages 7.5 dpc and 15.5 dpc, respectively. Thus, the significant increase in hepatic MDA level at the third studied embryonic age (15.5 dpc) in the exposed group could be explained by an increased oxidative stress status in rat embryos due to a significant decrease in the hepatic mitochondrial or cytosolic SOD relative activities as well as significant changes in their protein amounts. This result could be also referred to as a significant decrease in catalase mRNA expression and catalase antioxidant relative activities at this age of the development period^11^. The current study was the first one that investigated biochemical and molecular effects of prenatal exposure to GSM-EMR on the liver of rats at different embryonic ages. However, previous reports showed controversial postnatal effects of prenatal and/or postnatal EMR exposure on the gene expression, concentrations, and activities of different antioxidant enzymes such as SOD, catalase, and GPx. Diverse results were shown in these studies: a significant increase^34,41^, decrease^42,43^, or even non-significant effects^44^ in the level and/or the activities of these enzymes. Besides, it is important to mention that these results were affected by many factors such as the species, gender, ages, or studied organs in addition to the intensity, frequency, and duration of exposure to radiation, which makes difficult the comparison of the present study with the previous ones. Despite that, GSM-EMR exposure during the embryonic developmental period may induce an increase in the hepatic oxidative stress status at a specific embryonic age in rats due to the decrease in the cytosolic and mitochondrial SOD activity, as well as, catalase expression and activity, resulting in an increase in the MDA lipid peroxidation product level. The inevitable exposure to EMR radiations was shown to make changes at the level of gene expression, and increase apoptosis in different kinds of mammalian embryonic cells and tissues^45–48^. Exposure to these radiations was also shown to deregulate the cell cycle^49,50^, and lead to a chronic inflammation status that may result in different chronic disorders^51–53^. The findings of these previous studies lead us to study some other important factors at the molecular level such as the nuclear factor erythroïd 2-related factor 2 (Nrf-2). Nrf-2 is a key transcription factor that is normally present in the cytoplasm of various cell types^54^ and protects the cells by regulating the expression of genes related to anti-oxidative and anti-inflammatory responses, in addition to its conservative role as an anti-aging factor^55,56^. Nrf-2 is a multifunctional regulator that plays an important role in maintaining cellular homeostasis under stress conditions^57,58^. Due to its antioxidant capacity, Nrf-2 may be considered a potential therapeutic target in different types of diseases such as lung, chronic kidney diseases, and other diseases^57,59^. In the present study, Nrf-2 mRNA levels were significantly downregulated in the exposed group compared to the control animals at all studied embryonic ages. Moreover, the Nrf-2 protein level exhibited a non-significant change in the hepatic tissue of the exposed fetuses in comparison to the control ones at 19.5 dpc. Thus, exposure to EMR during the embryonic developmental period may result in a significant decrease in hepatic Nrf-2 mRNA expression that could be caused by a significant increase in Nrf-2 protein level or may be other causes at different ages. Further studies are required to determine the mechanisms and causes of these changes in rat embryos during exposure to the electromagnetic field in their prenatal period. Previous studies revealed a relationship between the activation and up-regulation of a serine or threonine-specific protein kinase (Akt), and the induction of Nrf-2 expression^60,61^. Akt1 (PKBα), Akt2 (PKBβ), and Akt3 (PKBγ) are the three main isoforms of Akt/PkB in mammalian genomes^62^. This protein kinase plays generally an important role in different cellular processes such as apoptosis, cell proliferation, glucose metabolism, and other cellular processes. Akt can also activate NF-kB and regulate Ikk leading to the transcription of pro-survival genes^63^. The Akt1 (PKBα), studied as a principal Akt isoform regulating apoptosis and involved in cell survival pathways, showed in its mRNA level expression a negative relation with that of the Nrf-2 gene over all the embryonic development period, where the exposed group showed a significant increase in Akt1 mRNA expression except a similar significant decrease to Nrf-2 at the age 15.5 dpc. These results contradict those of the previous studies except at a certain age where the significant reduction in Akt1 mRNA expression may indicate accelerated apoptosis in embryonic liver cells under radiation, compared to the control ones at the same age, and could explain the cause of multiple abortions in pregnant mothers with the difficulty of obtaining embryos at this stage especially, except after several attempts [personal observation]. Moreover, the significant reduction in Nrf-2 mRNA expression could be not correlated to the expression and activation of Akt1, but to other factors that should be determined during exposed embryos’ development. Nrf-2 activation increases cellular resistance to inflammatory challenges^64^. It may show an immunomodulatory effect on the immune system^64^ because of its presence at high levels in blood cells such as neutrophils, monocytes, and T and B cells^64,65^. The detection of a tissue injury by innate immune cells triggers the inflammation process to protect the host and initiate tissue repair^64^. Reactive oxygen species could act also as mediators of inflammation^64^. Thus, Nrf-2 activation plays an important role in the attenuation of inflammation-related to the production of ROS during the modulation of redox metabolism^64^. As a result of ROS generation, previous studies showed that Nrf-2 activation and translocation could coincide with the increase of the transcription of the pro-inflammatory cytokines such as ICAM-1^15^. ICAM-1, the intercellular adhesion molecule-1, is a cell surface glycoprotein and adhesion receptor involved in the leukocyte recruitment process from circulation to sites of inflammation. It is highly expressed in immune cells, epithelial, and endothelial cells after inflammatory stimulation, and has recently been shown to have a beneficial role in the resolution of inflammation and injury status^66^. Despite the decrease in ICAM-1 mRNA expression in the exposed embryos at all studied embryonic ages, EMR induced a significant increase in its expression at the late studied age (19.5 dpc). This could be explained by the absence of increased oxidative stress and inflammatory status in the exposed rats’ liver during the embryonic development period, which may be due to the influence of their mothers’ proteins produced during pregnancy and exposure. Pregnant mothers may try to protect their embryos from all radiation effects until a sensitive embryonic age (15.5 dpc) when later, separate cellular responses could begin in late-developed embryos.

In conclusion, our results showed that a daily permanent (24/24 h) GSM-EMW exposure, emitted from mobile phone relay antenna all over the prenatal development period of rat embryos could have certain effects on the hepatic oxidative stress, apoptotic, and inflammatory status at certain developmental stages. The prenatal exposure to these radiations could increase the free radicals production in liver tissues of rat embryos, and present an increased oxidative stress status resulting from the significant elevation of hepatic MDA level at the 15.5 embryonic age (15.5 dpc), with a significant decrease in some important antioxidant enzymes’ relative activities such as SOD in both cytosolic and mitochondrial fractions in addition to a significant reduction in catalase mRNA expression and relative activity. Moreover, a total significant decrease in Nrf-2, Akt1, and ICAM-1 mRNA expression in the liver of rat embryos at the only embryonic age 15.5 dpc may indicate an absence of an antioxidant, anti-apoptotic, and pro-inflammatory protective responses at this age, in addition to the presence of possible protective effects for rat embryos by their mothers from the increased hepatic oxidative stress (OS) status and its complications. Thus, further studies are required to detect the effects of this continuous prenatal exposure on the liver tissues of rat embryos, and to determine the mechanisms and origins of changes and protection of them from these radiations, in addition to the protection processes from expected or possible diseases or abortions.

### Supplementary Information

Supplementary Information.

## Data Availability

Data will be made available from the corresponding author on reasonable request.

## Abbreviations

GSM: Global system for mobile communication
EMW: Electromagnetic waves
GSM-EMF: Global system for mobile communication-like electromagnetic field
EMR: Electromagnetic radiation
RF: Radiofrequency signal
MHz: MegaHertz
OS: Oxidative stress
MDA: Malondialdehyde
CAT: Catalase
SOD: Superoxide dismutase
GPx: Glutathione peroxidase
NADPH: Nicotinamide adenine dinucleotide phosphate
mRNA: Messenger ribonucleic acid
ICAM-1: Intercellular adhesion molecule 1
Akt: Protein kinase
Nrf-2: Nuclear factor erythroid-2-related factor 2
h: Hours
NIH: National institutes of health
IRB: Institutional review board
°C: Degree celsius
Eeff: Effective field strength
V: Volt
m: Meter
cm: Centimeter
SAR: Specific absorption rate
kg: Kilogram
S: Siemens
ml: Milliliter
mg: Milligram
kg: Kilogram
U/ml: Units per milliliter
nmol: Nanomoles
min: Minutes
µl: Microliter
nm: Nanometer
μmol: Micromole
q-RT-PCR: Quantitative reverse transcription polymerase chain reaction
RNA: Ribonucleic acid
cDNA: Complementary deoxyribonucleic acid
β-actin: Beta-actin
TBP: TATA box binding protein
SDS: Sodium dodecyl sulfate
DPBS: Dulbecco's phosphate buffered saline
#: Number
dpc: Days post coitus
H_2_O_2_: Hydrogen peroxide
SPSS: Statistical package for the social sciences
*p*: *P*-Value (probability value)
GSH: Glutathione
GSSG: Oxidized glutathione
mitoSOD: SOD mitochondria fraction
cytoSOD: SOD cytosol fraction
CT: Threshold cycle
GAPDH: Glyceraldehyde 3-phosphate dehydrogenase
PKBα/Akt1: Protein kinase B alpha
PKBβ/Akt2: Protein kinase B beta
PKBγ/Akt3: Protein kinase B gamma
IKK: IkB kinase
NF-kB: Nuclear factor kappa B
ROS: Reactive oxygen species
SEM: Standard error of the mean
