# Supplementary material for: Impact of GSM-EMW exposure on the markers of oxidative stress in fetal rat liver
Source: Sci Rep. 2023 Oct 18;13:17806. doi: 10.1038/s41598-023-44814-z (PMC10584814; doi:10.1038/s41598-023-44814-z)
Supplement: Supplementary file 1 — Supplementary Information. [file 41598_2023_44814_MOESM1_ESM.pdf]

Impact of GSM-EMW exposure on the markers of oxidative stress in fetal rat liver

Mariam Salameh[2], Sukaina Zeitoun-Ghandour[1], Lina Sabra[1], Ahmad Daher[3], Mahmoud Khalil[2,4] and Wissam H. Joumaa[1]

[1]: Rammal Hassan Rammal Research Laboratory, PhyToxE research group, Lebanese University, Faculty of Sciences (V), Nabatieh, Lebanon

[2]: Department of Biological Sciences, Faculty of Science, Beirut Arab University, Beirut, Lebanon

[3]: Rammal Hassan Rammal Research Laboratory, ATAC research group, Lebanese University, Faculty of Sciences (I), Hadat, Lebanon.

[4]: Molecular Biology Unit, Department of Zoology, Faculty of Science, Alexandria University, Egypt

Effect of exposure to GSM-EMW on Nrf-2 and GAPDH protein expression by western blotting in liver of rat fetuses at 19.5 dpc

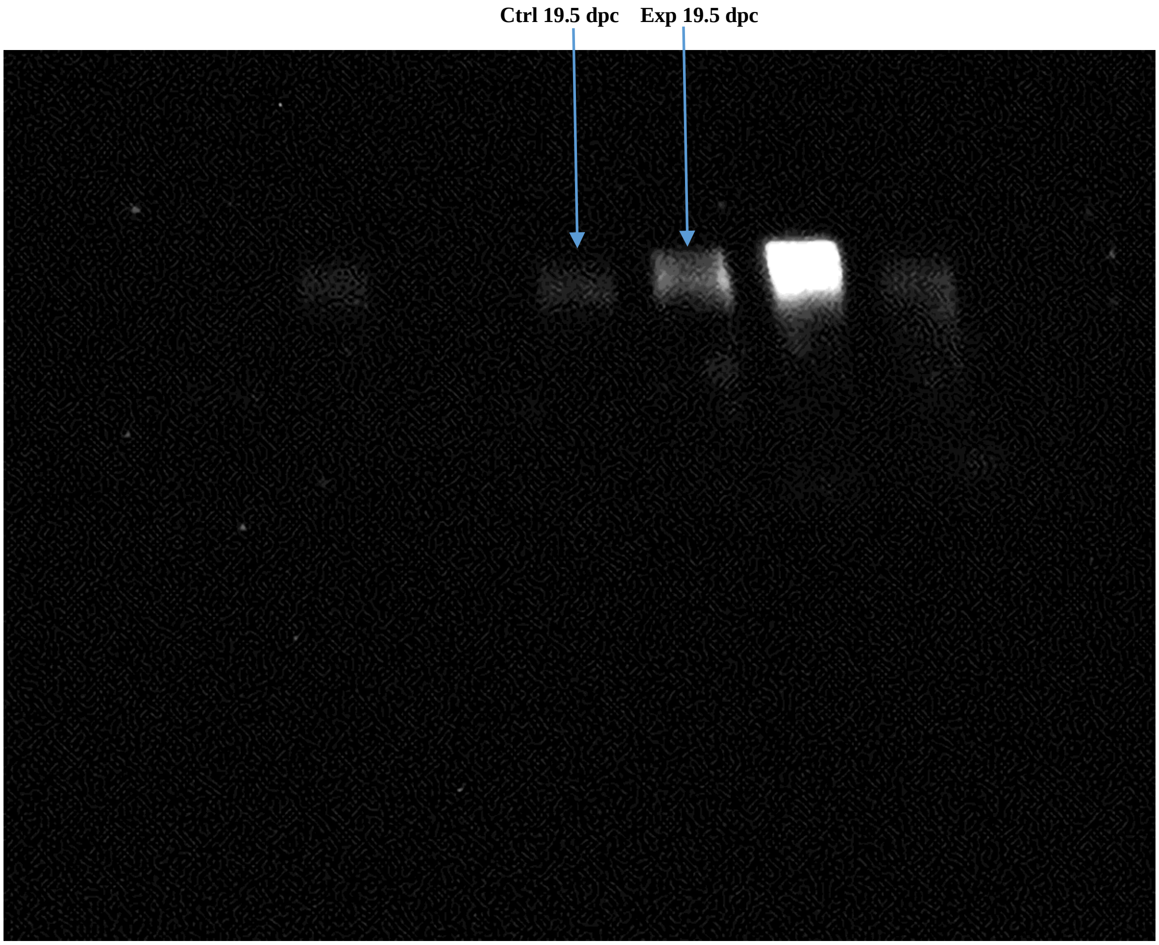

(a)

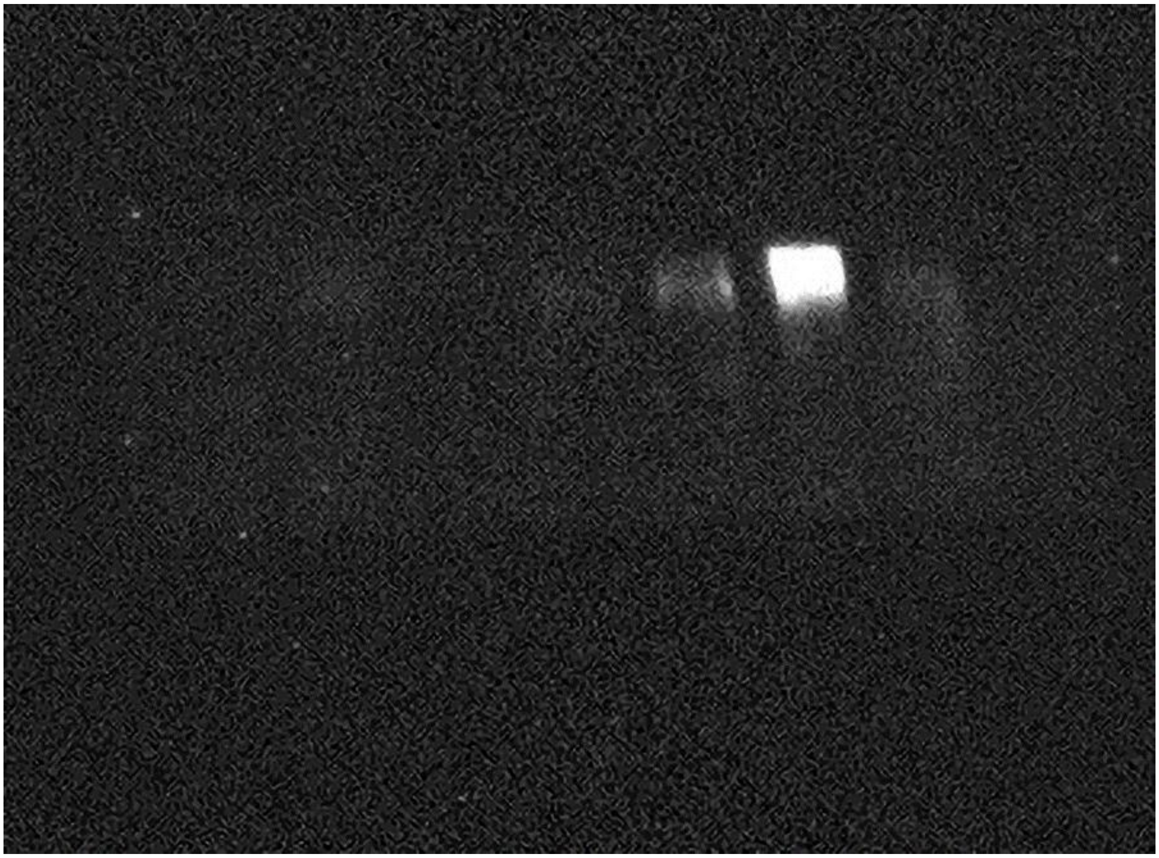

(a1)

(a2)

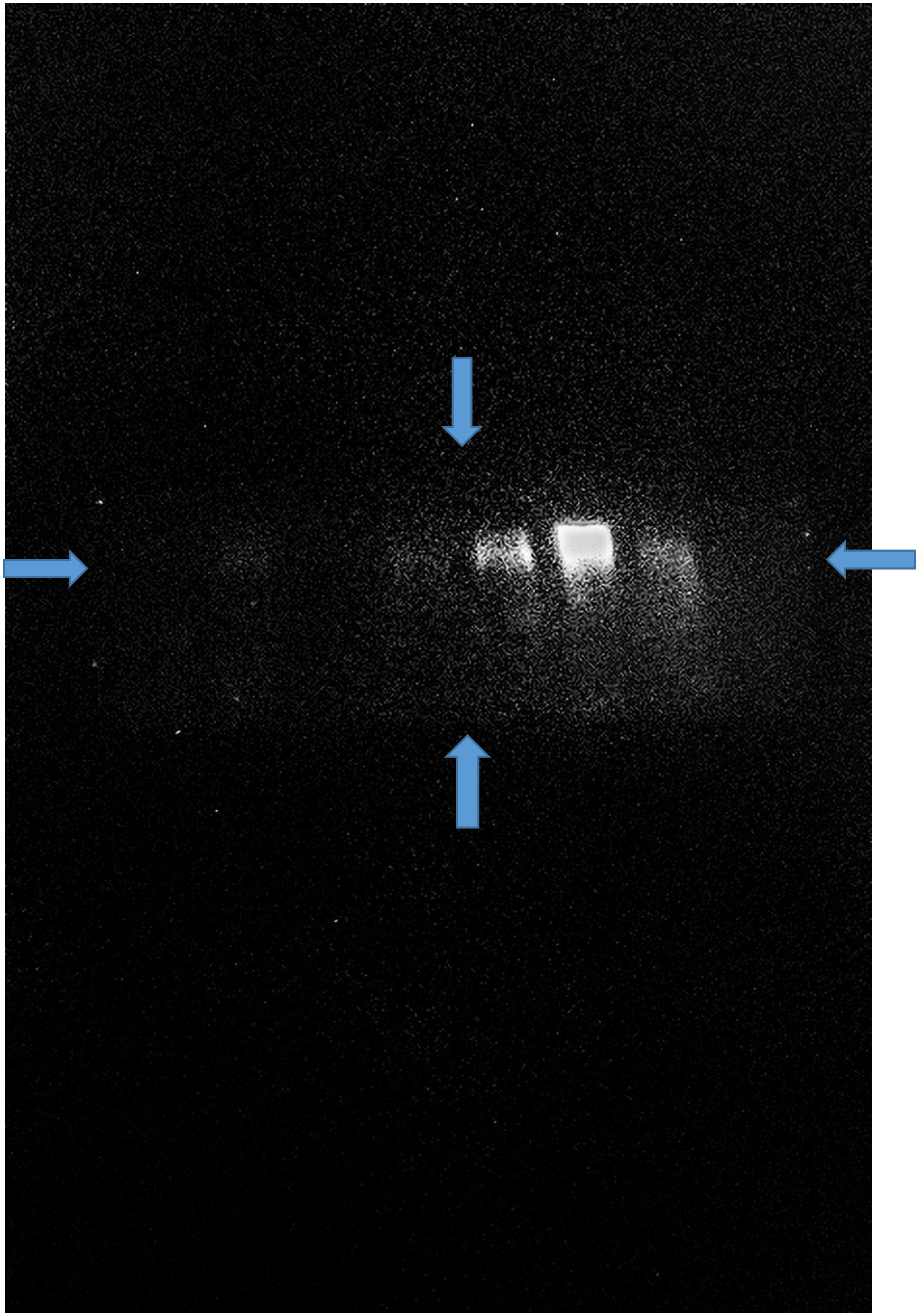

(a2)

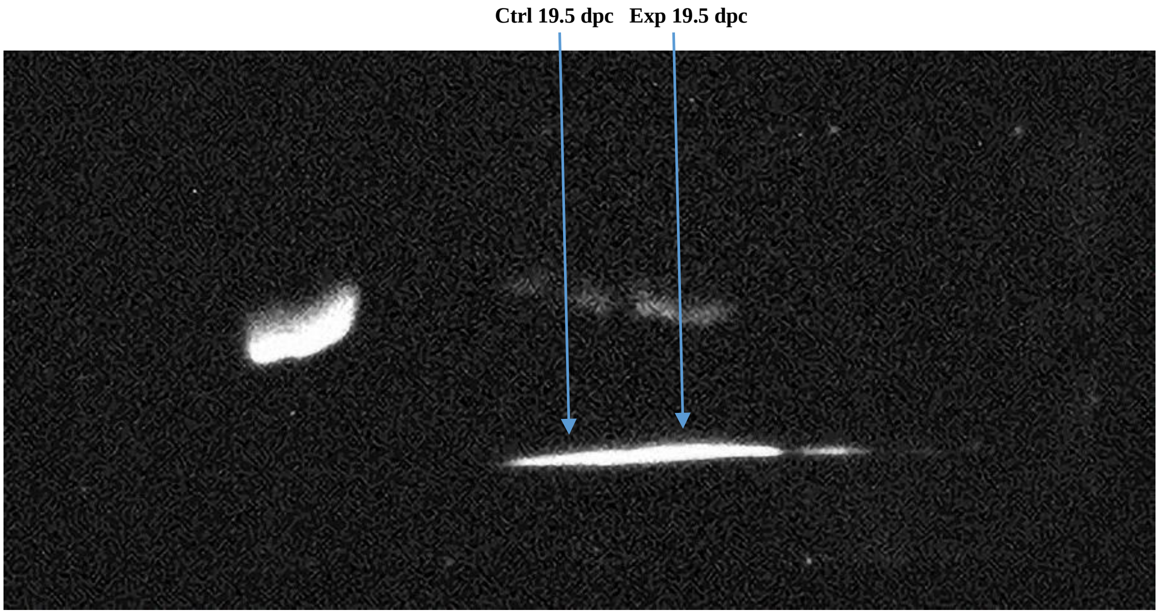

(b)

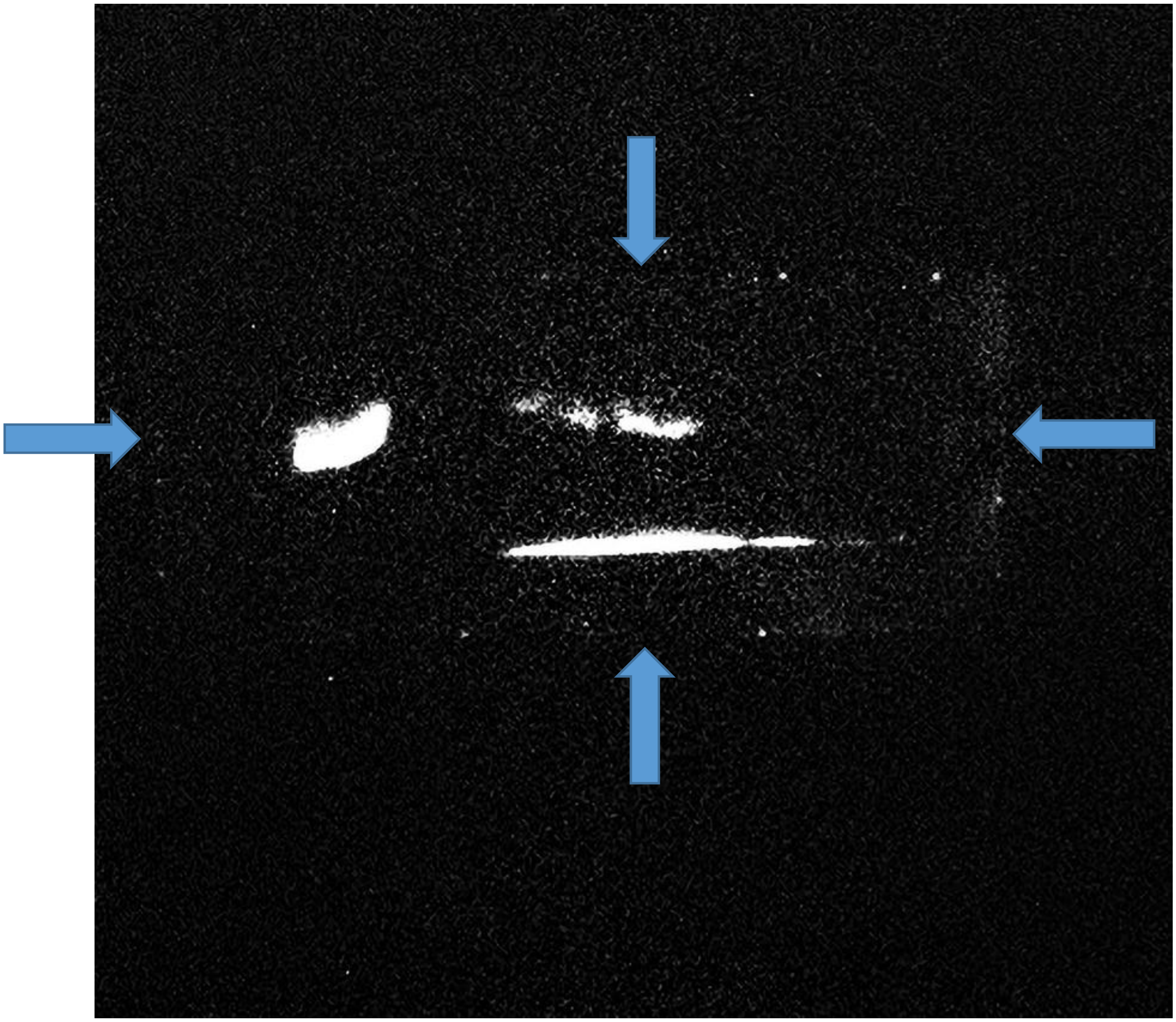

(b1)

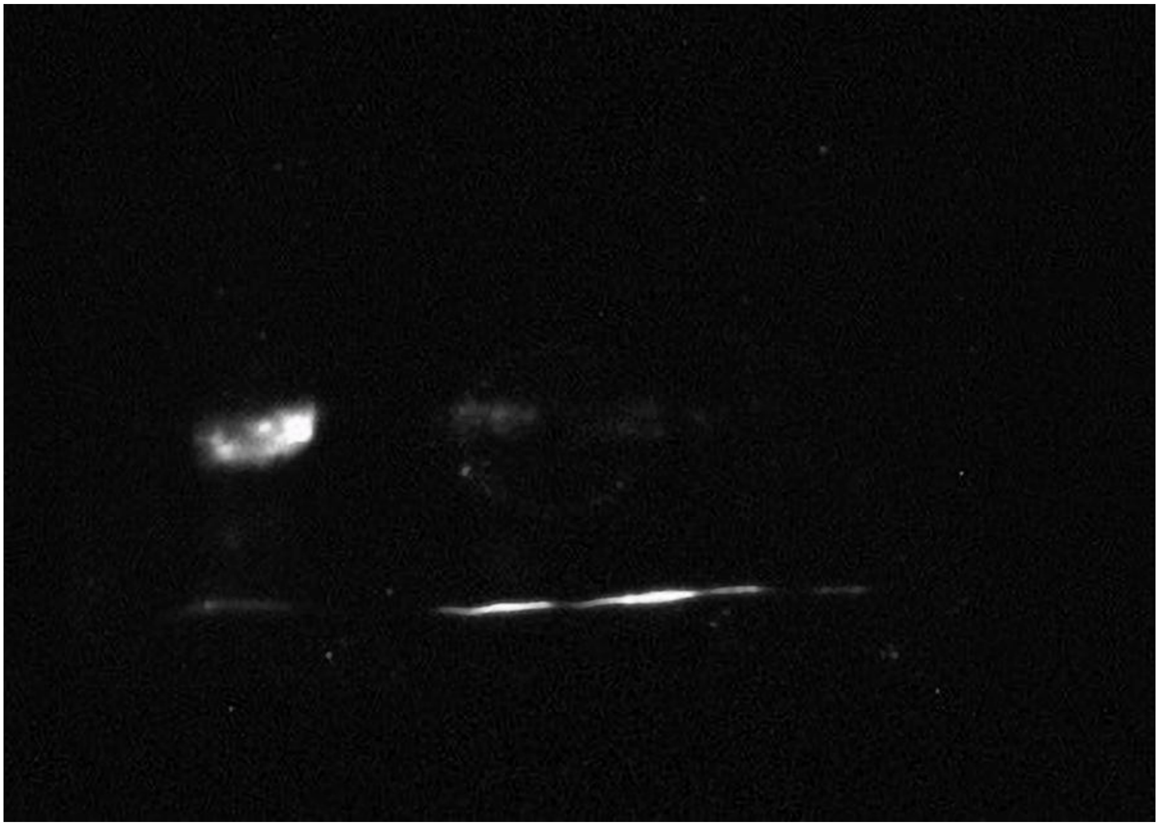

(b2)

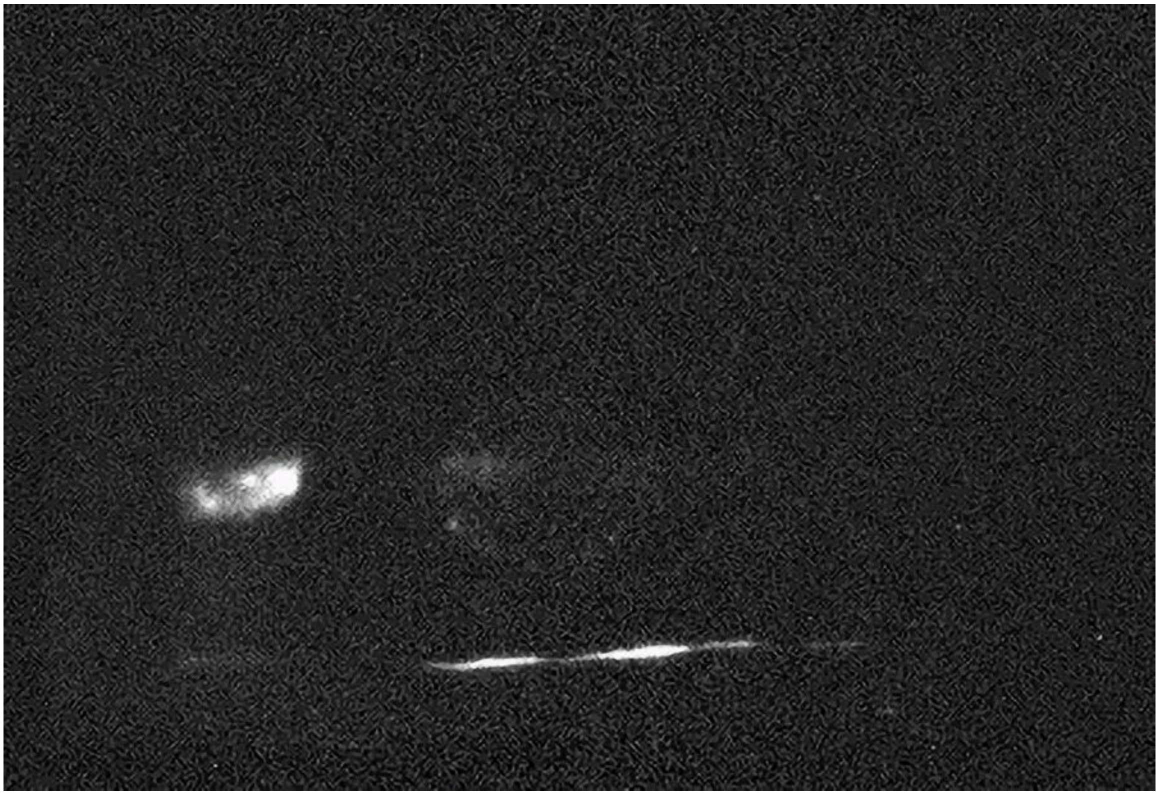

(b3)

Fig.5(original) Effects of electromagnetic radiation (EMR) exposure on nuclear factor erythroid-2-related factor 2 (Nrf-2) protein expression. (a) and (b) are the original gels containing the western blotting results for nuclear factor erythroid-2-related factor 2 (Nrf-2) and glyceraldehyde 3-phosphate dehydrogenase (GAPDH) reference protein in the liver of rat fetuses (n=3) at 19.5 dpc in both control (Ctrl 19.5 dpc) and exposed group (Exp 19.5 dpc), respectively. N.B: (a1, a2), and (b1, b2, b3) were added to make the edges of the membranes representing the blots shown in (a), and (b) respectively, more visible and identified. Original images of full-length blots cannot be provided because these blots/membranes were cut depending only on the areas containing the desired proteins, prior the step of hybridisation with antibodies.
